# An Anti-FOD Method Based on CA-CM-CFAR for MMW Radar in Complex Clutter Background

**DOI:** 10.3390/s20061635

**Published:** 2020-03-14

**Authors:** Xiaoqi Yang, Kai Huo, Jianwei Su, Xinyu Zhang, Weidong Jiang

**Affiliations:** 1 Graduate School, National University of Defense Technology, Changsha 410073, China; yangxiaoqinudt@sina.com; 2 College of Electronic Science and Technology, National University of Defense Technology, Changsha 410073, China; sujianwei99@hotmail.com (J.S.); zhangxinyu9011@163.com (X.Z.); jwd2232@vip.163.com (W.J.)

**Keywords:** foreign object debris, millimeter-wave radar, clutter map, constant false alarm rate, clutter edge, variability index

## Abstract

Traditional constant false alarm rate (CFAR) methods have shown their potential for foreign object debris (FOD) indication. However, the performance of these methods would deteriorate under the complex clutter background in airport scenes. This paper presents a threshold-improved approach based on the cell-averaging clutter-map (CA-CM-) CFAR and tests it on a millimeter-wave (MMW) radar system. Clutter cases are first classified with variability indexes (VIs). In homogeneous background, the threshold is calculated by the student-t-distributed test statistic; under the discontinuous clutter conditions, the threshold is modified according to current VI conditions, in order to address the performance decrease caused by extended clutter edges. Experimental results verify that the chosen targets can be indicated by the t-distributed threshold in homogeneous background. Moreover, effective detection of the obscured targets could also be achieved with significant detectability improvement at extended clutter edges.

## 1. Introduction

According to MOOG Aircraft Group, one of the biggest airplane component-makers in the world, over 66% of airport emergencies are related to foreign object debris (FOD) on runways [1]. Urgent requirements for reliable FOD inspection have been indicated to the aviation industry.

Among applied systems, radars perform better than electro-optical devices especially in inclement conditions [1,2]. Existing systems (such as Tarsier [1], operating in 94.5GHz) have shown that microwave radars can provide high resolutions to defense metal, stones, concrete, or even plastics with small radar cross-section (RCS) on runways and air operations area (AOA) surfaces [3] (pp. 5–6). Moreover, some other high-resolution radars have been successively developed and testified by simulations and outfield experiments, operating around wide-range single frequencies (e.g., 76.5 GHz [4], 77 GHz [5,6], 78 GHz [7], and 96 GHz [2]). In Ref. [8], a multi-frequency study was presented, focusing both on the comparative measurement of asphalt clutter and on the RCS of typical FOD targets across a wide spectral band [8]. 

High-resolution radars have become the primary sensors of airport surveillance [9,10]. In conjunction with other types of sensors to provide an integrated data fusion, advanced surface movement guidance and control systems (A-SMGCSs) have been developed [11], as the most advanced AOA control concept in the world [12]. With the sustained technical support of manufacturer (such as THALES Group in [13]), A-SMGCSs keep providing controllers with improved situational awareness, to enhance surface airport movement and enable advanced tower cab functionality. 

Radar-based FOD surveillance is always challenged by heavy land clutter in practice, thus constant false alarm rate (CFAR) algorithms possess the potential to support anti-FOD radars. 

There are two common methods of implementing CFAR:

In the first method, the detector outputs are calculated by proper background estimation, such as cell-averaging (CA) [14,15,16], the greatest or smallest option (GO or SO) [17], ordered statistic (OS) [18,19], from nearby cells only in space domain. Such procedures may suffer from spatial heterogeneity, leading to poor detection probability or excessive false alarms. Hence some methods [20,21,22] with robustness were proposed, but further improvements are still required. Recent investigations [23,24] focus on detectability improvement to the low-altitude, slow-speed, small targets, considering complex heterogeneous background, which aims at real applications.

Another is known as clutter-map (CM) technique [25,26], which exploits temporal stationarity of clutter background rather than in spatial domain. In detail, the estimation of the background power (or amplitude) is obtained by averaging previous returns of each map cell in some certain manner. However, the performance will degrade when targets enter the resolution cells or persist during several scanning periods. To reduce target self-masking [27,28] and false alarms [28], improvements have been put forward under the Gaussian [27,29] or various non-Gaussian background (e.g., exponentially distributed clutter [27], log-normal clutter [28], K-distributed clutter [30], and Weibull clutter [31]).

In many cases, a single CFAR processors can hardly meet the complex radar operation environment. Thus, the concept of variability indexes (VIs) were introduced, to account for both homogeneous and heterogeneous clutters. It performs intelligent detection using composite approach, based on four basis CFARs (CA-, SO-, GO- and OS-CFAR) [32,33,34]. 

In recent years, CFAR methods have been involved in radar-based FOD detection. Ref. [35] provided the theoretical basis for CFAR engineering application against FOD. In [36], the authors proposed two former detection information- (FDI-) CFARs. The performance outperformed in homogeneous or partially homogeneous clutter than in heterogeneous conditions, which exposed the detection challenge brought by topography discontinuity. The plane technique of CM-CFAR was first introduced for FOD detection in [37]. It was validated in relatively low signal-to-clutter ratio (SCR) situation, such as AOA surfaces in airport. Aiming at multi-FOD, the authors in [38] proposed a Trimmed-Mean CM-CFAR method based on OS. Several of the large samples in a reference window were trimmed to tolerate interfering targets. By employing feature extraction and support vector domain description, experimental results showed that it could not only detect but also classify foreign items and false alarms [39]. 

For practical application, improvements with robustness are still required, especially when various scattering surfaces are involved. Giving full consideration to the complex clutter background in AOA scenes, a hybrid method based on CA-CM-CFAR is proposed in this paper. In addition, we also present experimental results acquired at 78.5GHz in a pavement scenario. Compared to previous work, the contributions of our work are summarized as follows:

1. The clutter edges caused by background discontinuities, which may impede the traditional CFAR performance is first being considered. In previous research [35,36,37,38,39], scenarios of homogeneous runway were considered most commonly.
2. According to VIs, we modify the threshold near clutter edges, bringing convenience to acquire threshold. Moreover, it is decoupled with the scene knowledge.
3. The feasibility for MMW radar is verified by an experimental system.

The rest of this paper contains four main sections. In Section 2, the theoretical background is introduced. Section 3 addresses the adaptive method based on the CA-CM-CFAR, in homogeneous or clutter edge conditions. Accordingly, the evaluation indexes of performance are proposed. In Section 4, the experiment set-up at 78.5 GHz is described. Finally, conclusions are drawn according to the experiment results in Section 5.

## 2. Theoretical Basis

In this section, the basic theory about CA-CM-CFAR and VIs are introduced. The CM, where the runway and side lawns are covered, is depicted according to a typical AOA scene. Compared with the Nitzberg CM-CFAR, another CFAR involving adjacent map cells in background estimation is introduced. Moreover, VIs are computed to indicate current clutter background within the reference window, which play as the basis of threshold selection.

### 2.1. Map Cell Division

**Figure 1:**
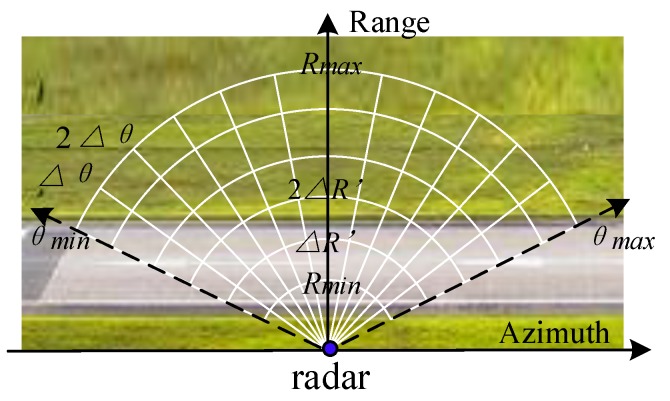
The scene is divided into several resolution cells, and the background level is saved as clutter-map (CM) matrix and updating with the radar sensor scanning.

As in Figure 1, a side-looking radar is equipped on a rotary platform and scanning:

Two terrain surfaces, lawns and runways, are involved. The antenna energy is concentrated within the main beam whose width is $\theta_{b}$ in azimuth. With the beam scanning, the radar coverage is limited from $R_{min}$ to $R_{max}$ in range, and from $\theta_{min}$ to $\theta_{max}$ in azimuth. Thus, the background is divided into resolution cells sized ΔR×Δθ.$\;\Delta R^{\prime }$ indicates the projected ΔR. The clutter level of each cell (intensity or amplitude) is saved in matrix form, known as the static CM. With one-by-one scanning, the CM is updating at an efficiency. In general cases, the efficiency is control within (0,1).

### 2.2. CA-CM-CFAR

Classical CM-CFAR algorithms are investigated in temporal domain. The fluctuant CM of each scanning is levelling off to the theoretical scattering power with the increasing of iteration times. There are two CM-CFAR techniques according to different estimations of clutter level at cell under test (CUT).

**Figure 2:**
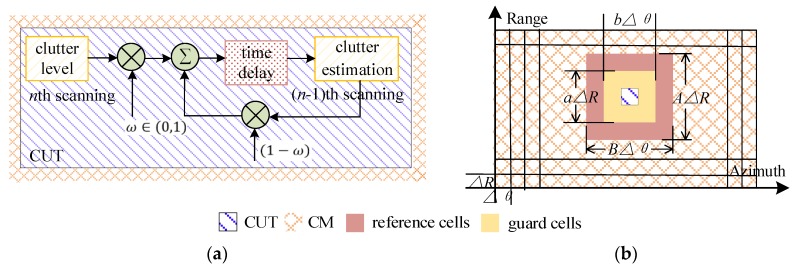
The estimations of clutter level at the cell under test (CUT) during CM-constant false alarm rate (CFAR) processing: (**a**) The ‘point’ technique of Nitzberg CM-CFAR; (**b**) The plane technique used in the hybrid CM-CFAR.

One is known as the Nitzberg technique [25] (see Figure 2a), or ‘point’ technique of CM-CFAR, which means only CUT clutter power is involved. The background estimation is acquired by averaging previous clutter power in CUT, at an iteration rate of ω∈(0,1).

Another is known as the plane technique [27,29]. The spatial samples from a bunch of map cells are grouped in a reference window, and iteratively filtered on a scan-by-scan basis, under an assumption of clutter model. As Figure 2b shows, a reference window sized AΔR×BΔθ (A, B are required to be odd and no less than 3) is sliding on the CM, where ab guard units (also known as the protection units) are contained to prevent power spread of the extended targets. Thus, there are (AB−ab) map cells involved in background estimating.

The CM-CFAR with plane technique is employed in this paper for FOD indication, due to higher detection probability than Nitzberg CM-CFARs, under the same SCR condition and false alarm requirement [40] (pp. 29–30). The CUT background is achieved by CA; thus, it is mentioned as CA-CM-CFAR in the later sections.

### 2.3. VI Indicator

VIs could describe current clutter conditions dynamically under complex background, which are commonly used as indicators to select CFAR method (e.g., CA, SO, GO or OS) adaptively.

**Figure 3:**
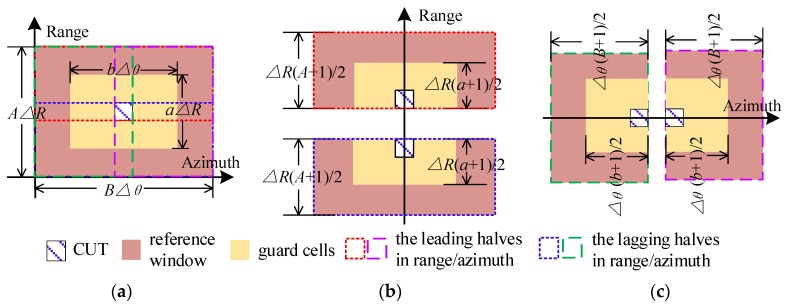
Reference window division for variability indexes (VIs): (**a**) Such a reference window sized *A*Δ*R* × *Bθ* is divided into two pairs of halves highlighted by different dotted lines; (**b**) Division in range, each half is (A+1)ΔR/2 wide; (**c**) Division in azimuth, with two (B+1)θ/2-width halves.

Take a reference window sized AΔR×BΔθ (ΔR and Δθ denote the resolutions) as the example, ab guard units are removed from the VI calculation. First, we divide the reference window into two pairs of equal halves (as Figure 3) in range and azimuth, respectively. Hence a Gaussian statistic $Y^{i,j}\left(n\right)\sim N\left(\mu_{Y},\sigma_{Y}^{2}\right)$ with mean $\mu_{Y}$ and variance $\sigma_{Y}^{2}$, is introduced to denote the clutter background at *n*th scanning, where i and j indicate the range and azimuth positions on the CM. To achieve relatively stable clutter levels, K integrations are carried out. The integrated result is expressed as another Gaussian statistic $X^{i,j}\left(n\right)$, satisfying $X^{i,j}\left(n\right)\sim N\left(K\mu_{Y},K\sigma_{Y}^{2}\right)\;$. Calculate the ratio between leading and lagging reference halves, the range VI is expressed by $V_{ran}^{i_{0},j_{0}}$:

(1) $$V_{ran}^{i_{0},j_{0}}(n)=\frac{\sum_{i=i_{0}}^{i_{0}+\frac{A-1}{2}}\sum_{j=j_{0}-\frac{B-1}{2}}^{j_{0}+\frac{B-1}{2}}X^{i,j}(n)-\sum_{i=i_{0}}^{i_{0}+\frac{a-1}{2}}\sum_{j=j_{0}-\frac{b-1}{2}}^{j_{0}+\frac{b-1}{2}}X^{i,j}(n)}{\sum_{i=i_{0}}^{i_{0}}{}_{-\frac{A-1}{2}}\sum_{j=j_{0}-\frac{B-1}{2}}^{j_{0}+\frac{B-1}{2}}X^{i,j}(n)-\sum_{i=i_{0}-\frac{A-1}{2}}^{i_{0}}\sum_{j=j_{0}-\frac{b-1}{2}}^{j_{0}+\frac{b-1}{2}}X^{i,j}(n)}$$

Similarly, the azimuthal VI $V_{azi}^{i_{0},j_{0}}$ is computed as

(2) $$V_{azi}^{i_{0},j_{0}}(n)=\frac{\sum_{i=i_{0}-\frac{A-1}{2}}^{i_{0}+\frac{A-1}{2}}\sum_{j=j_{0}}^{j_{0}+\frac{B-1}{2}}X^{i,j}(n)-\sum_{i=i_{0}-\frac{a-1}{2}}^{i_{0}+\frac{a-1}{2}}\sum_{j=j_{0}}^{j_{0}+\frac{b-1}{2}}X^{i,j}(n)}{\sum_{i=i_{0}-\frac{A-1}{2}}^{i_{0}+\frac{A-1}{2}}\sum_{j=j_{0}-\frac{B-1}{2}}^{j_{0}}X^{i,j}(n)-\sum_{i=i_{0}-\frac{a-1}{2}}^{i_{0}+\frac{a-1}{2}}\sum_{j=j_{0}-\frac{b-1}{2}}^{j_{0}}X^{i,j}(n)}$$

Both $V_{ran}^{i_{0},j_{0}}$ and $V_{azi}^{i_{0},j_{0}}$ are obtained by the clutter level ratio between reference halves. In general, the lawn clutter levels are much higher than runway especially in low-grazing conditions. It is easy to find that: $V_{ran}^{i_{0},j_{0}}\left(\mathrm{dB}\right)$ and $V_{azi}^{i_{0},j_{0}}\left(\mathrm{dB}\right)$ will be kept around zero in the cases of homogeneous reference windows (only grass or runway surface is involved), whereas the non-zero VIs demonstrate the presence of background discontinuities in the leading or lagging half. Considering about clutter fluctuation in practice, the VI conditions are relaxed as in Table 1:

Please note that the limitations $\xi_{r}$ and $\xi_{a}$ are required small and positive. 

## 3. CFAR-Based Detector

**Figure 4:**
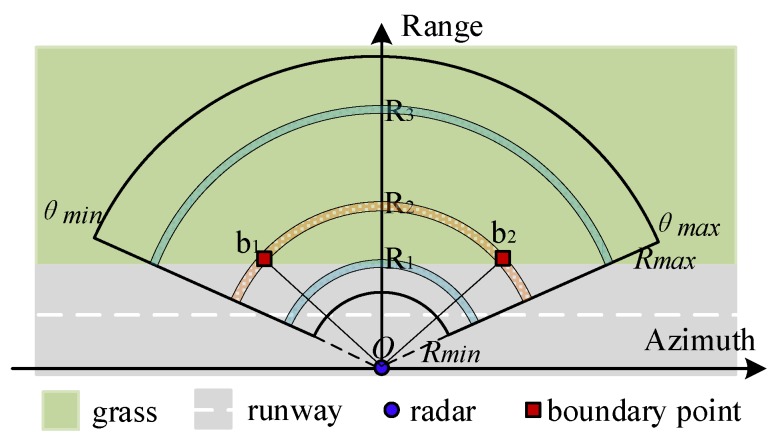
Complex background conditions are considered: homogeneous runway or grass surface is involved in range gate R_1_ or R_3_; clutter edges are generated by terrain discontinuity within the same range gate, as demonstrated by R_2_.

As Figure 4 demonstrates, three range gates are taken as the examples, named $R_{1}$ to $R_{3}$. The ground clutter is believed homogeneous at those range gates, which are closer than $R_{1}$ or further than $R_{3}$, because only the concrete/asphalt runway or lawn surface is involved. As for the others, more surface conditions must be taken into account, which brings sharp clutter changes at the terrain boundaries (indicated by $b_{1}$ and $b_{2}$, located at range gate $R_{2}$). 

A CFAR-based detector is presented in this section which adaptively selects threshold according to the VI conditions. In addition, the indexes of performance evaluation are given.

### 3.1. Homogeneous Clutter Conditions

The clutter levels are assumed to have statistically independent and identical distribution (IID) within a uniform reference window. When IID is satisfied, the clutter mean and variance are generally estimated based on their statistical properties (probability densities are the most commonly used). At *n*th scanning, we first estimate the mean ${\bar{X}}^{i_{0},j_{0}}\left(n\right)$ and variance $V^{i_{0},j_{0}}\left(n\right)$ of clutter amplitude at$\;\left(i_{0},\;j_{0}\right)$ CUT as

(3a) $${\bar{X}}^{i_{0},j_{0}}\left(n\right)=\frac{1}{AB-ab}\left[\overset{i_{0}+\frac{A-1}{2}}{\underset{i=i_{0}-\frac{A-1}{2}}{\sum }}\overset{j_{0}+\frac{B-1}{2}}{\underset{j=j_{0}-\frac{B-1}{2}}{\sum }}X^{i,j}\left(n\right)-\overset{i_{0}+\frac{a-1}{2}}{\underset{i=i_{0}-\frac{a-1}{2}}{\sum }}\overset{j_{0}+\frac{b-1}{2}}{\underset{j=j_{0}-\frac{b-1}{2}}{\sum }}X^{i,j}\left(n\right)\right]$$

(3b) $$\begin{array}{c} V^{i_{0},j_{0}}\left(n\right)=\frac{1}{AB-ab-1}[\overset{i_{0}+\frac{A-1}{2}}{\underset{i=i_{0}-\frac{A-1}{2}}{\sum }}\overset{j_{0}+\frac{B-1}{2}}{\underset{j=j_{0}-\frac{B-1}{2}}{\sum }}{\left(X^{i,j}\left(n\right)-{\bar{X}}^{i_{0},j_{0}}\left(n\right)\right)}^{2}\\ -\overset{i_{0}+\frac{a-1}{2}}{\underset{i=i_{0}-\frac{a-1}{2}}{\sum }}\overset{j_{0}+\frac{b-1}{2}}{\underset{j=j_{0}-\frac{b-1}{2}}{\sum }}{\left(X^{i,j}\left(n\right)-{\bar{X}}^{i_{0},j_{0}}\left(n\right)\right)}^{2}] \end{array}$$

${\bar{X}}^{i_{0},j_{0}}\left(n\right)$ subjects to Gaussian distribution with variance $K\sigma_{Y}^{2}/\left(AB-ab\right)$ and mean $K\mu_{Y}\;$while $V^{i_{0},j_{0}}\left(n\right)$ obeys a modified Chi-square distribution with (AB−ab−1) degrees of freedom (DOFs) (the deductions are given in Appendix A), which are expressed as

(4a) $${\bar{X}}^{i_{0},j_{0}}\left(n\right)\sim N\left(K\mu_{Y},\frac{K\sigma_{Y}^{2}}{\left(AB-ab\right)}\right)$$

(4b) $$V^{i_{0},j_{0}}\left(n\right)\sim \frac{K\sigma_{Y}^{2}}{\left(AB-ab-1\right)}\chi^{2}\left(AB-ab-1\right)$$

With one-by-one scanning, ${\bar{X}}^{i_{0},j_{0}}$ and $V^{i_{0},j_{0}}$ are updating at ω∈(0,1). This procedure is realized in the form of iterative filter. Suppose ${\bar{X}}_{f}^{i_{0},j_{0}}\left(n\right)$ and $V_{f}^{i_{0},j_{0}}\left(n\right)$ play the filter output, both statistics are generated by previous ${\bar{X}}^{i_{0},j_{0}}$ and $V^{i_{0},j_{0}}$. Such expressions are obtained:

(5a) $${\bar{X}}_{f}^{i_{0},j_{0}}\left(n\right)=\left(1-\omega \right){\bar{X}}_{f}^{i_{0},j_{0}}\left(n-1\right)+\omega {\bar{X}}^{i_{0},j_{0}}\left(n\right)$$

(5b) $$V_{f}^{i_{0},j_{0}}\left(n\right)=\left(1-\omega \right)V_{f}^{i_{0},j_{0}}\left(n-1\right)+\omega V^{i_{0},j_{0}}\left(n\right)$$

Equations above are initialized by ${\bar{X}}_{f}^{i_{0},j_{0}}\left(1\right)={\bar{X}}^{i_{0},j_{0}}\left(1\right)$ and $V_{f}^{i_{0},j_{0}}\left(1\right)=V^{i_{0},j_{0}}\left(1\right)$. ${\bar{X}}_{f}^{i_{0},j_{0}}\left(n\right)$ and $V_{f}^{i_{0},j_{0}}\left(n\right)$ share similar distributions to ${\bar{X}}^{i_{0},j_{0}}\left(n\right)$ and $V^{i_{0},j_{0}}\left(n\right)$ (according to the details in Appendix A). Referring to (4a) and (4b), we introduce ϖ=(2−ω)/ω to simplify the statistical models:

(6a) $${\bar{X}}_{f}^{i_{0},j_{0}}\left(n\right)\;\sim N\left(K\mu_{Y},K\sigma_{Y}^{2}/\varpi \left(AB-ab\right)\right)$$

(6b) $$V_{f}^{i_{0},j_{0}}\left(n\right)\sim \frac{K\sigma_{Y}^{2}}{\varpi \left(AB-ab-1\right)}\chi^{2}\left(\varpi \left(AB-ab-1\right)\right)$$

Thus, the decisions are deduced as (7a) and (7b), where T indicates the detection threshold.

(7a) $${\bar{X}}^{i_{0},j_{0}}\left(n\right)\begin{array}{c} \geq T{\bar{X}}_{f}^{i_{0},j_{0}}\left(n-1\right);\;H_{1}\\ <T{\bar{X}}_{f}^{i_{0},j_{0}}\left(n-1\right);\;H_{0} \end{array}$$

(7b) $$V^{i_{0},j_{0}}\left(n\right)\begin{array}{c} \geq TV_{f}^{i_{0},j_{0}}\left(n-1\right);\;H_{1}\\ <TV_{f}^{i_{0},j_{0}}\left(n-1\right);\;H_{0} \end{array}$$

$H_{1}$ (or $H_{0}$) denotes the presence (or absence) of targets. In fact, the test statistic can be improved by combining (7a) and (7b). Thus, $X_{E}^{i_{0},j_{0}}\left(n\right)$ is put forward as the test statistics based on the difference between ${\bar{X}}^{i_{0},j_{0}}\left(n\right)$ and ${\bar{X}}_{f}^{i_{0},j_{0}}\left(n-1\right)$ (both are Gaussian):

(8) $$X_{E}^{i_{0},j_{0}}\left(n\right)={\bar{X}}^{i_{0},j_{0}}\left(n\right)-{\bar{X}}_{f}^{i_{0},j_{0}}\left(n-1\right)\begin{array}{c} \geq T\sqrt{V_{f}^{i_{0},j_{0}}\left(n-1\right)};\;H_{1}\\ <T\sqrt{V_{f}^{i_{0},j_{0}}\left(n-1\right)};\;H_{0} \end{array}$$

The false alarm rate $P_{fa}$ is decided by

(9) $$\begin{array}{ll} P_{fa}=P\{X_{E}\left(n\right)> & T\sqrt{V_{f}\left(n-1\right)}|H_{0}\}\\ & =\int_{0}^{\infty }\int_{0}^{T\sqrt{V_{f}\left(n-1\right)}}f_{1}\left(X_{E}\left(n\right)\right)d\left(X_{E}\left(n\right)\right)f_{2}(V_{f}\left(n-1\right))d(V_{f}\left(n-1\right))\\ & =\int_{0}^{\infty }F_{1}\left(T\sqrt{V_{f}\left(n-1\right)}\right)f_{2}(V_{f}\left(n-1\right))d(V_{f}\left(n-1\right)) \end{array}$$

where $f_{1}\left(\cdot \right)$ indicates the probability density function (PDF) of $X_{E}\left(n\right)$. $F_{1}\left(\cdot \right)$ represents the cumulative distribution function (CDF) of $f_{1}\left(\cdot \right)$. Meanwhile, the multiple integration in (9) is hard to solve in an analytical form, consisting of the Chi-square PDF $f_{2}\left(\cdot \right)$ and Normal CDF $F_{1}\left(\cdot \right)$.

Referring to (4a) and (6a), we can get the following conclusion:

(10) $$\frac{{\bar{X}}^{i_{0},j_{0}}\left(n\right)-{\bar{X}}_{f}^{i_{0},j_{0}}\left(n-1\right)}{\sqrt{\left[\varpi \left(AB-ab\right)+1\right]K\sigma_{Y}^{2}/\left[\varpi \left(AB-ab\right)\right]}}\sim N\left(0,1\right)$$

Therefore, we introduce a statistic $D_{E}^{i_{0},j_{0}}\left(n\right)$:

(11) $$\begin{array}{ll} D_{E}^{i_{0},j_{0}} & \left(n\right)=\frac{{\bar{X}}^{i_{0},j_{0}}\left(n\right)-{\bar{X}}_{f}^{i_{0},j_{0}}\left(n-1\right)}{\sqrt{\left[\varpi \left(AB-ab\right)+1\right]K\sigma_{Y}^{2}/\left[\varpi \left(AB-ab\right)\right]}}\cdot \sqrt{\frac{K\sigma_{Y}^{2}\varpi \left(AB-ab-1\right)}{V_{f}^{i_{0},j_{0}}\left(n-1\right)\varpi \left(AB-ab-1\right)}}\\ & \;=\frac{{\bar{X}}^{i_{0},j_{0}}\left(n\right)-{\bar{X}}_{f}^{i_{0},j_{0}}\left(n-1\right)}{\sqrt{\left[\varpi \left(AB-ab\right)+1\right]/\left[\varpi \left(AB-ab\right)\right]}\cdot \sqrt{V_{f}^{i_{0},j_{0}}\left(n-1\right)}}\\ & \;=\frac{{\bar{X}}^{i_{0},j_{0}}\left(n\right)-{\bar{X}}_{f}^{i_{0},j_{0}}\left(n-1\right)}{\varpi^{\prime }\cdot \sqrt{V_{f}^{i_{0},j_{0}}\left(n-1\right)}}=\frac{X_{E}^{i_{0},j_{0}}\left(n\right)}{\varpi^{\prime }\cdot \sqrt{V_{f}^{i_{0},j_{0}}\left(n-1\right)}}\sim t\left(\varpi \left(AB-ab-1\right)\right) \end{array}$$

$D_{E}^{i_{0},j_{0}}\left(n\right)$ obeys the Student-t distribution with ϖ(AB−ab−1) DOFs, where $\varpi^{\prime }=\sqrt{\left[\varpi \left(AB-ab\right)+1\right]/\left[\varpi \left(AB-ab\right)\right]}$. Thereupon the threshold T should be modified as

(12) $$T_{E}=T/\varpi^{\prime }$$

Thus, (9) can be rewritten as

(13) $$P_{fa}=P\left\{D_{E}\left(n\right)>T_{E}|H_{0}\right\}=\int_{T_{E}}^{\infty }f_{3}\left(D_{E}\left(n\right)\right)d\left(D_{E}\left(n\right)\right)=1-F_{3}\left(T_{E}\right)$$

where $f_{3}\left(\cdot \right)$ indicates the t distribution PDF with a CDF $F_{3}\left(\cdot \right)$. $P_{fa}$ is strictly constant and independent of the clutter level. Thereupon, it is convenient to solve $T_{E}$ as

(14) $$T_{E}=1-F_{3}^{-1}\left(P_{fa}\right)$$

when $P_{fa}$ keeps constant. $F_{3}^{-1}\left(\cdot \right)$ is the inverse function of $F_{3}\left(\cdot \right)$. 

### 3.2. Clutter Edge Conditions

**Figure 5:**
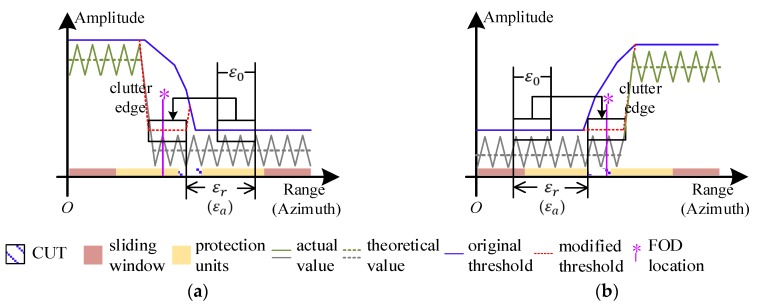
A veiled target near the sharp clutter edge is indicated by the modified threshold, which is intercepted from the homogeneous runway side when (**a**)$\;V_{ran}^{i_{0},j_{0}}\left(\mathrm{dB}\right)<-\xi_{r}$ or $V_{azi}^{i_{0},j_{0}}\left(\mathrm{dB}\right)<-\xi_{a}$; (**b**) $V_{ran}^{i_{0},j_{0}}\left(\mathrm{dB}\right)>\xi_{r}$ or $V_{azi}^{i_{0},j_{0}}\left(\mathrm{dB}\right)>\xi_{a}$.

Targets with small RCSs may be masked by the inflated threshold near background discontinuities. The performance of CA-CFAR detectors would deteriorate in these cases. Thus, a threshold modification is proposed as shown in Figure 5:

The clutter echoes are signified by green and gray lines fluctuating around the theoretical level (denoted by the corresponding dotted lines). A FOD target near the clutter edge (where $\left|V_{ran}^{i_{0},j_{0}}\left(\mathrm{dB}\right)\right|>\xi_{r}$ or $\left|V_{azi}^{i_{0},j_{0}}\left(\mathrm{dB}\right)\right|>\xi_{a}$) is veiled by the threshold of CA-CFAR (represented by the blue lines), thus we introduce $\varepsilon_{r}$ (or $\varepsilon_{a}$) in range (or azimuth) domain to indicate the shift distance (in the direction from grass surface to runway) where the $\varepsilon_{0}$-length threshold is intercepted, to replace the counterpart on the runway side. Hence the modified threshold $T_{mod}$ is highlighted by red. To sum up, $T_{E}$ is lowered for better detectability around the terrain discontinuities. 

### 3.3. Performance Evaluation

The detection probability $P_{d}$ is used to evaluate CA-CM-CFAR in homogeneous clutter:

(15) $$\begin{array}{ll} P_{d}\;=\;P\{D_{E}(n)> & T_{E}|H_{1}\}\;=\;\int_{T_{E}}^{\infty }f_{3}(D_{E}(n)-\sqrt{\lambda_{x}}{\left[1+\varpi (AB-ab)\right]}^{-\frac{1}{2}})d(D_{E}(n))\\ & =\int_{T_{E}}^{\infty }f_{3}(D_{E}(n)-\sqrt{\lambda_{x}}{\left[1+\varpi (AB-ab)\right]}^{-\frac{1}{2}})d(D_{E}(n)\\ & -\sqrt{\lambda_{X}}{\left[1+\varpi (AB-ab)\right]}^{-\frac{1}{2}})=\int_{T_{E}{}^{\prime }}^{\infty }f_{3}(D_{E}{}^{\prime }(n))d(D_{E}{}^{\prime }(n))=1-F_{3}(T_{E}{}^{\prime }) \end{array}$$

$T_{E}{}^{\prime }=T_{E}-\sqrt{\lambda_{X}}{\left[1+\varpi \left(AB-ab\right)\right]}^{-\frac{1}{2}}$, $D_{E}{}^{\prime }\left(n\right)=D_{E}\left(n\right)-\sqrt{\lambda_{X}}{\left[1+\varpi \left(AB-ab\right)\right]}^{-\frac{1}{2}}$, where $\lambda_{X}$ denotes the SCR after K noncoherent integrations. 

Around the clutter edges, $T_{E}$ is modified to $T_{mod}$ according to current VIs, which suggests that $P_{d}$ is difficult to solve, due to the complex clutter is hard to be depicted by any analytical CDF. Therefore, we present the indicator η(i,j) as

(16) $$\eta \left(i,j\right)=T_{E}\left(i,j\right)/T_{mod}\left(i,j\right)\;$$

to evaluate the improvement of detectability, which is required by a smallest detectable target.

## 4. Experiment Set-Up

To confirm the performance, an MMW radar system is carried out. A concrete pavement scenario with similar terrains (a concrete pavement and the side) to runway scenes is employed, aiming at five chosen FOD targets.

### 4.1. Radar Sensor

**Figure 6:**
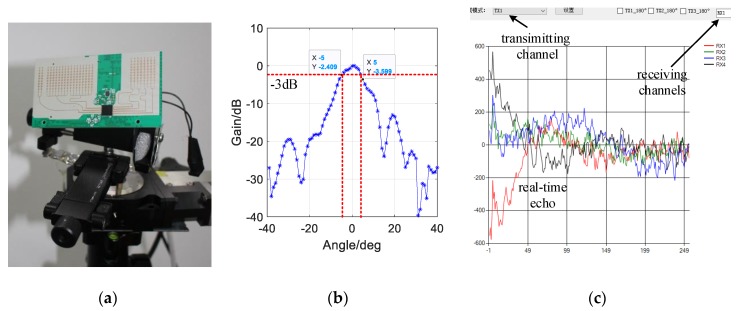
Some details of the radar system: (**a**) The planar folded reflect-array antenna; (**b**) The measured beam pattern in horizon, with 3 dB aperture of 10 deg; (**c**) The real-time data when selecting the first transmitting channel, preliminarily processed by the analyzing software.

The frequency modulated-continuous wave (FMCW) is generated and modulated in linear triangle manner, which allows 1.5 GHz bandwidth from 77.75 GHz to 79.25 GHz. There are 256 samples during the 25.6 μs chirp, where the pulse repetition interval (PRI) is 0.1 μs. A cosec shaped beam (in Figure 6b) is realized by a planar folded reflect-array antenna (95 mm × 52 mm, see Figure 6a) with three transmitting and four receiving channels. The time domain data is recorded and performed by the analyzing software as in Figure 6c. The real-time echo from a pair of transmitting/receiving channels, TX1 and RX1, is selected and processed by Fast Fourier Transform (FFT). The radar carrier is controlled to determine the scanning range and spatial sampling step, for spatial sampling and CM updating. Some details about the MMW radar are provided in Table 2.

### 4.2. Test FOD Targets

**Figure 7:**
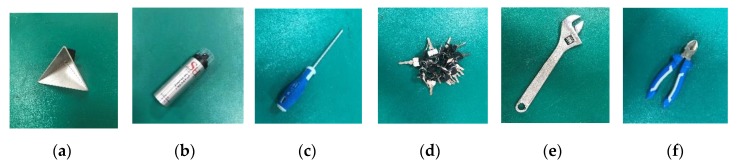
The calibration object and foreign object debris (FOD)targets: (**a**) Calibration object: a trihedral corner reflector; (**b**) Target 1: an aluminum aerosol bottle; (**c**) Target 2: a screwdriver; (**d**) Target 3: a string of keys; (**e**) Target 4: a metal spanner; (**f**) Target 5: a pair of pliers with rubber handles.

Six objects are considered in our investigation. As in Figure 7a, a trihedral corner reflector sized 13.5 cm × 13.5 cm × 13.5 cm is used as the reference object to achieve the beam pattern. The chosen targets are: an aluminum aerosol bottle (Target 1, in Figure 7b), the height is 19.3 cm and the radius is 2.5 cm; a 21 cm screwdriver with 10.5 cm rubber handle (Target 2, shown in Figure 7c); a string of 24 keys, each one is 5 cm (Target 3, see Figure 7d); a 24.8 cm metal spanner (Target 4, see Figure 7e) and a pair of pliers (Target 5, in Figure 7f), which is 16 cm long, with 4 cm metallic part. All targets are placed in front of the radar. It should be noted that the asymmetric targets are placed with their long sides facing the antennas. 

### 4.3. Operating Scene

**Figure 8:**
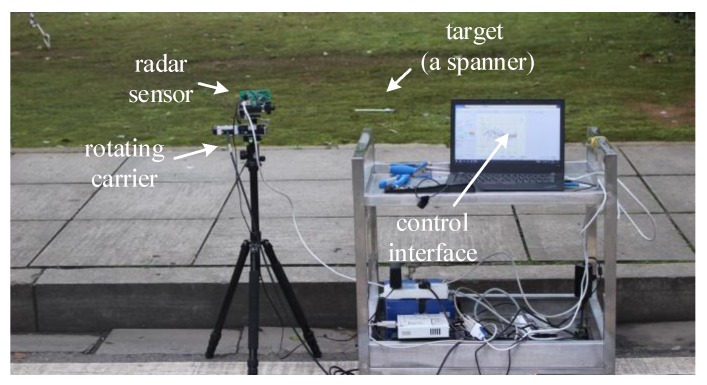
The scene containing a concrete pavement and the side lawn is scanned by the radar system.

Figure 8 shows the integral system, composed of the planar folded antenna, an FMCW radar module, and a control interface displayed by a PC screen. The radar sensor is placed by a 0.7 m height holder. The rotation of the whole system is carried out on the horizontal plane with a motor under the antenna.

The radar carrier is located at one side of a concrete pavement, which is 2.1 m wide. The adjacent lawn is also covered by the elevation beam. The clutter cells are expecting to be reliable only within the main lobes in both horizon and elevation. Please note that the lawn and pavement share the same horizon level. 

## 5. Results and Discussion

To validate the detection method above, three experiments are carried out at 78.5 GHz. In Experiment 1, two objects are considered on the homogeneous background. As for Experiment 2 and 3, three and four targets are involved respectively, some are masked near the clutter edges. The detection results are given and discussed, against the target masking. 

Table 3 provides some conditions of this hybrid CFAR:

There are some other details to clarify: the real-time echo is processed by two-dimension FFT with 128 bins in range-azimuth domain, thus the theoretical radar coverage is from 0.1 m to 12.8 m. Hanning window is used in FFT processing.

- Experiment 1

There are two objects, Target 2 (the screwdriver) on the grassland and 3 (the string of keys) on the concrete pavement are concerned in this experiment. See Table 4, the listed positions indicate their geometric centers.

**Figure 9:**
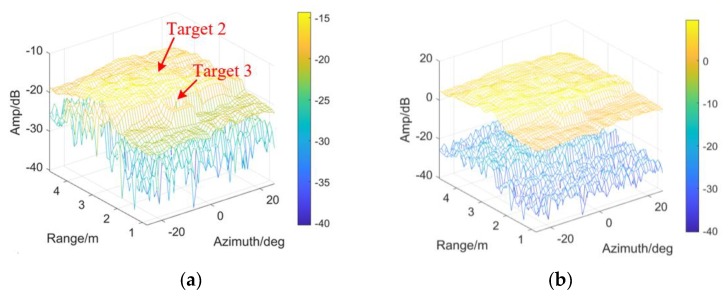
The outputs of CA-CM-CFAR detectors by employing (**a**) Student-t test statistics; (**b**) Gaussian test statistics.

As widely known, student-t models approximate Gaussian distributions mathematically with DOF increasing. The outputs of CFAR processor by the student-t test statistics are shown in Figure 9a, compared with the Normal statistics in Figure 9b. Both objects are missed by the Normal threshold, which demonstrates that the test statistics can be hardly depicted by Gaussian distribution. 

**Figure 10:**
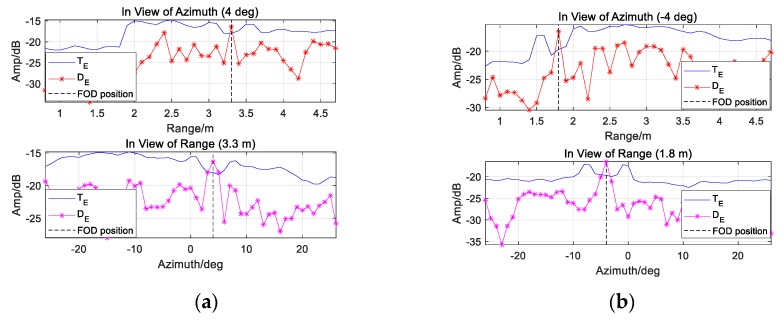
The results of CA-CM-CFAR processor are shown in view of range and azimuth, aiming at (**a**) Target 2; (**b**) Target 3.

Figure 10 presents the result in Figure 9a from the perspective of the indicated target positions, range, and azimuth included. Both objects are detected agrees with the real positions. Such a conclusion is drawn, the proposed CFAR works to those targets with larger RCSs, in the uniform background.

**Figure 11:**
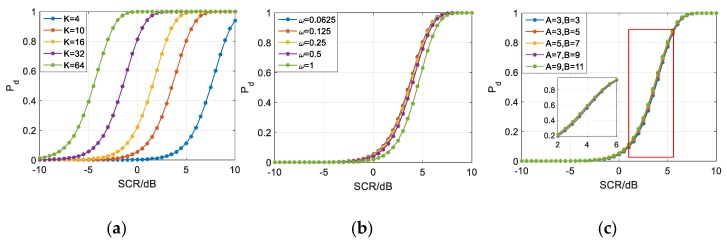
Theoretical *P_d_* of CA-CM-CFAR of CA-CM-CFAR in homogeneous background: (**a**) A=7, B=9, ω=0.0625; (**b**) A=7, B=9, K=10; (**c**) ω=0.0625 and K=10.

In the cases of homogeneous background, the detection performance evaluated by $P_{d}$ is presented in Figure 11. All the plots refer to $P_{fa}\;$=$\;10^{-6}$.

Figure 11a illustrates the plots of $P_{d}$ versus SCR in different K conditions, when A=7, B=9, and ω=0.0625. Better performance would be obtained with larger K under the same SCR condition. Figure 11b shows that the detector performance after ten integrations could be slightly improved by increasing ω, when A and B remain the same. Please note that the case ω=1 denotes the invalidation of iterative CM. In different A or B conditions, the plots in the third subfigure depict the performance variation when K=10, ω=0.0625. Even the partial enlarged drawing suggests that $P_{d}$ is only marginally improved under larger AB.

- Experiment 2

The real positions (all still denote the target geometric centers) of Target 1 (an aluminum aerosol bottle), 3 (a string of keys) and 5 (a pair of pliers with rubber handles) are displayed in Table 5. 

**Figure 12:**
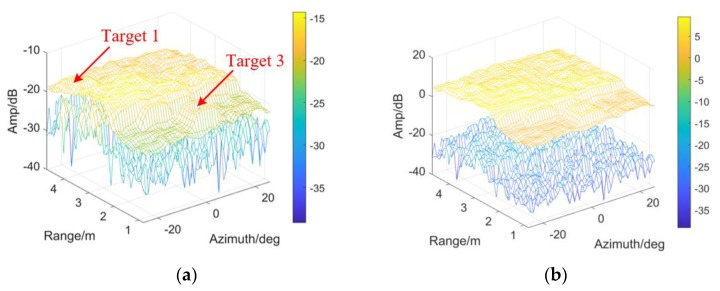
The outputs of CA-CM-CFAR by employing (**a**) Student-t test statistics; (**b**) Gaussian test statistics.

All three items are veiled by the Normal threshold in Figure 12b while Target 1 and 3 are clearly indicated by the t-distributed detector in subfigure a. $D_{E}\left(n\right)\sim t\left(\varpi \left(AB-ab-1\right)\right)$ is verified. 

However, the threshold obeying t distribution misses Target 5 near the terrain boundary (around 2.2 m, calculated as $\sqrt{H^{2}+W^{2}}$), which has smaller RCS (see Figure 7f, mainly because of the rubber handles) than the aluminum Target 1. Thus, threshold modification is required according to Section 3.2, since the reference map cells around the obscured object is believed heterogeneous when terrain boundaries are involved.

**Figure 13:**
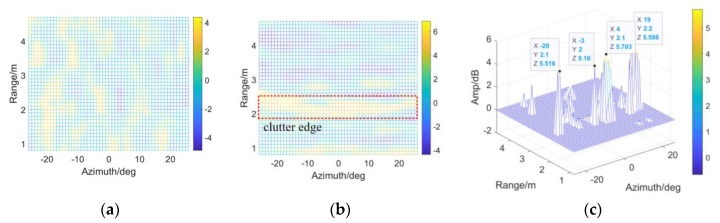
Detectability are improved according to the VIs: (**a**) VI in azimuth; (**b**) VI in range; (**c**) *η* of the scene.

In Figure 13a, there is not significant terrain boundary in azimuth, from −30 deg to 30 deg. In Figure 13b, an extended clutter edge around 2.2 m is brought by the dramatic change between different scattering surfaces, as highlighted by the VIs much larger than zero dB. According to Figure 13a,b, the detectability improvement, denoted by η, is displayed in Figure 13c. Considering about the clutter fluctuations,$\;\varepsilon_{0}$ is relaxed to the length of interception where $\left|V_{ran}^{i_{0},j_{0}}\;\left(\mathrm{dB}\right)\right|\geq 2$ and $\left|V_{azi}^{i_{0},j_{0}}\;\left(\mathrm{dB}\right)\right|\geq 3$, thus $T_{mod}$ could be obtained as in Figure 5 when $\varepsilon_{r}=\left(A-1\right)/2$ and $\varepsilon_{a}=\left(B-1\right)/2$. Significant improvement (>5 dB) is achieved, especially between the pavement and lawn.

**Figure 14:**
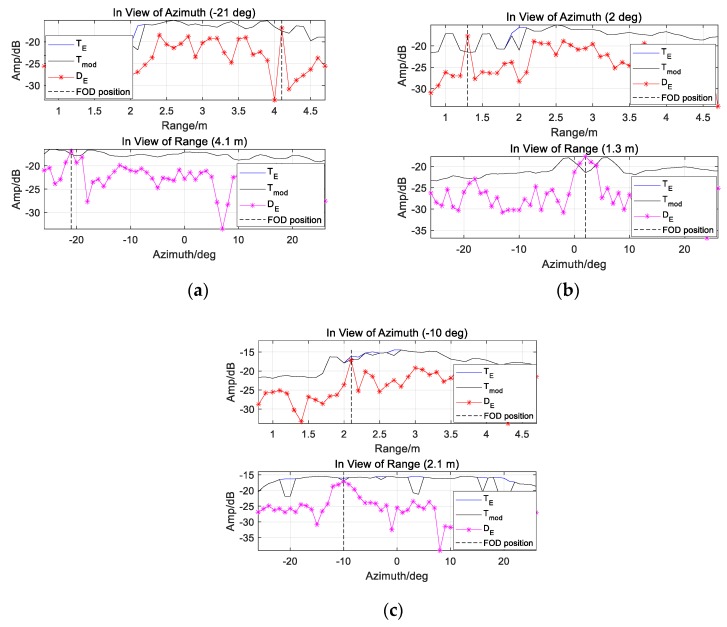
The results of CA-CM-CFAR detector are shown in view of range and azimuth, aiming at (**a**) Target 1; (**b**) Target 3; (**c**) Target 5, by employing $T_{E}$ and $T_{mod}$ respectively.

Figure 14 provides $T_{E}$ and $T_{mod}$ in view of range and azimuth.$\;T_{mod}$ around Target 1 and 3 are almost equal to $T_{E}$. We also notice that the veiled Target 5 is indicated by $T_{mod}$, which shows that $T_{mod}$ has better performance than $T_{E}$.

- Experiment 3

Target 1 (the aluminum bottle), 2 (the screwdriver), 4 (the spanner) and 5 (the pliers) are concerned in the third experiment. Their positions are given in Table 6.

Notice that the first two targets may be disturbed by the extended background discontinuities. The other targets are located at the homogeneous pavement or lawn.

**Figure 15:**
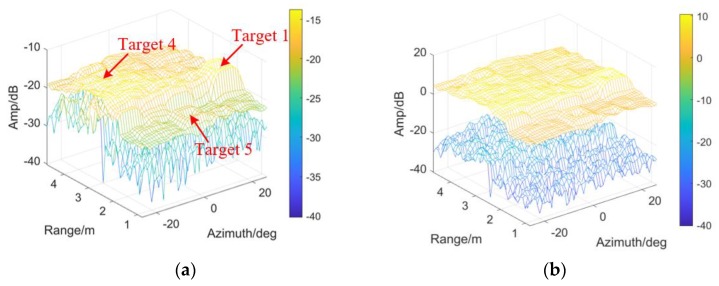
The outputs of CFAR detectors by employing (**a**) Student-t test statistics; (**b**) Gaussian test statistics.

In Figure 15, the Normal threshold is employed to compare with the student-t threshold. The same conclusion as Figure 9 and Figure 12 is obtained: $D_{E}$ is believed t-distributed. 

**Figure 16:**
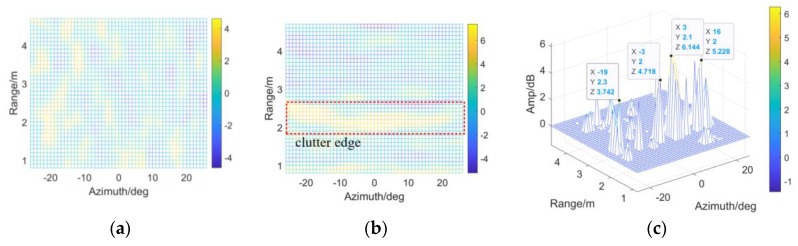
Detectability improvement and the VIs: (**a**) VI in azimuth; (**b**) VI in range; (**c**) *η* of the scene.

$\varepsilon_{r}$, $\varepsilon_{a}$ and $\varepsilon_{0}$ remain unchanged, the threshold is modified for detectability improvement according to the VIs in Figure 16a,b. As the evaluation index, η is shown in Figure 16c. More than 3 dB improvement could be obtained at the clutter edge especially in range, around 2.2 m. To sum up, the modified detector has the potential to overcome the target masking, when sharp changes are involved on the CM.

**Figure 17:**
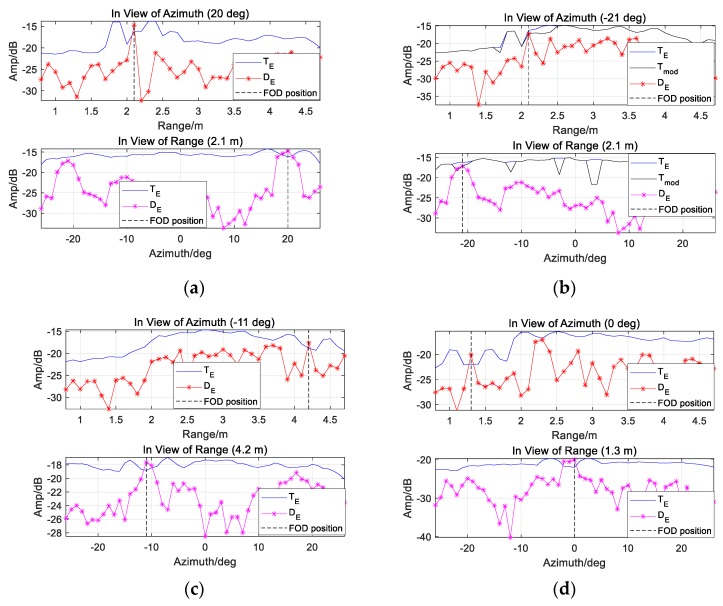
The results of CA-CM-CFAR detector are shown in view of range and azimuth, aiming at (**a**) Target 1; (**b**) Target 2; (**c**) Target 4; (**d**) Target 5, by employing $T_{E}$ or $T_{mod}$ respectively.

As shown in the following figures in view of range and azimuth, all the four targets are indicated, by employing $T_{E}$ or $T_{mod}$. See Figure 17a,b, $T_{E}$ could detect Target 1 rather than Target 2 (with smaller RCS). Meanwhile, the CFAR-based detector with $T_{mod}\;$(denoted by the black lines) indicate veiled Target 2 effectively, no matter in range or azimuth.

## 6. Conclusions

CFAR algorithms for radar-based FOD detection deserves more attention for many compelling advantages under all time and all weather. However, the performance of these methods would deteriorate under the complex clutter background in airport scenes. This paper presented a threshold-improved approach based on a cell-averaging clutter-map (CA-CM-) CFAR and tested it on a millimeter-wave (MMW) radar system. Clutter cases were first classified with variability indexes (VIs). In homogeneous background, the threshold was calculated by the student-t-distributed test statistic; under the discontinuous clutter conditions, the threshold was modified according to current VI conditions, to address the performance decreasing caused by extended clutter edges. Experimental results verified that the chosen targets can be indicated by the t-distributed threshold in homogeneous background. Moreover, effective detection of the obscured targets could also be achieved with significant detectability improvement at extended clutter edges.

Nevertheless, target extension in azimuth, as a result of the horizontal aperture, need to be restrained to avoid false alarms. We also admit that some other debris with smaller RCSs are worthy of concerns, aiming at better detectability. In addition, future works will test in situ the radar system on an airport runway, which is essential to develop A-SMGCSs.

## Figures and Tables

**Table 1:** VI conditions and the corresponding surface cases within the reference window.

VI Conditions	$V_{ran}^{i_{0},j_{0}}\left(\mathrm{dB}\right)>\xi_{r}$ $/V_{azi}^{i_{0},j_{0}}\left(\mathrm{dB}\right)>\xi_{a}$	$\left|V_{ran}^{i_{0},j_{0}}\left(\mathrm{dB}\right)\right|<\xi_{r}$ $/\left|V_{azi}^{i_{0},j_{0}}\left(\mathrm{dB}\right)\right|<\xi_{a}$		$V_{ran}^{i_{0},j_{0}}\left(\mathrm{dB}\right)<-\xi_{r}$ $/V_{azi}^{i_{0},j_{0}}\left(\mathrm{dB}\right)<-\xi_{a}$
**Leading half**	lawn	lawn	runway	runway
**Lagging half**	runway	lawn	runway	lawn

**Table 2:** Specifications of the 78.5 GHz radar.

Frequency Band	Antenna Gain	Ranging Method	Horizontal 3 dB Aperture	PRI
77.75–79.25 GHz	13.11 dBi	FMCW	10 deg	0.1 μs
**Bandwidth**	**Transmitting power**	**Frequency modulation**	**Angular step**	**Chirp length**
1.5 GHz	10 dBm	Linear triangle	1 deg	25.6 μs

**Table 3:** Some conditions of cell-averaging (CA-)CM-CFAR.

Parameter	Symbol	Value	Parameter	Symbol	Value
carrier height	H	0.7 m	pavement width	W	2.1 m
range coverage	$\left[R_{min},R_{max}\right]$	[0.1,12.8] m	angular scope	$\left[\theta_{min},\theta_{max}\right]$	[−30,30] deg
range resolution	ΔR	0.1 m	scanning step	Δθ	1 deg
protection length	a/b	3/5	reference length	A/B	7/9
updating efficiency	ω	0.0625	false alarm rate	$P_{fa}$	$10^{-6}$

**Table 4:** Target positions in Experiment 1.

Target	Target 2	Target 3
Range	3.3 m	1.8 m
Azimuth	4 deg	−4 deg

**Table 5:** Target positions in Experiment 2.

Target	Target 1	Target 3	Target 5
Range	4.1 m	1.3 m	2.1 m
Azimuth	−21 deg	−2 deg	−10 deg

**Table 6:** Target positions in Experiment 3.

Target	Target 1	Target 2	Target 4	Target 5
Range	2.1 m	2.1 m	4.2 m	1.3 m
Azimuth	20 deg	−21 deg	−11 deg	0 deg

## Appendix A

According to $X^{i,j}\left(n\right)\sim N\left(K\mu_{Y},K\sigma_{Y}^{2}\right)$, the mean of (AB−ab) reference samples ${\bar{X}}^{i_{0},j_{0}}\left(n\right)$ (see (4a)) is also Normal distributed, which is deduced as

(A1) $$\begin{array}{ll} E\left[{\bar{X}}^{i_{0},j_{0}}\left(n\right)\right]= & E\left\{\frac{1}{AB-ab}\left[\overset{i_{0}+\frac{A-1}{2}}{\underset{i=i_{0}-\frac{A-1}{2}}{\sum }}\overset{j_{0}+\frac{B-1}{2}}{\underset{j=j_{0}-\frac{B-1}{2}}{\sum }}X^{i,j}\left(n\right)-\overset{i_{0}+\frac{a-1}{2}}{\underset{i=i_{0}-\frac{a-1}{2}}{\sum }}\overset{j_{0}+\frac{b-1}{2}}{\underset{j=j_{0}-\frac{b-1}{2}}{\sum }}X^{i,j}\left(n\right)\right]\right\}\\ & \;=\frac{ABE\left[X^{i,j}\left(n\right)\right]}{AB-ab}-\frac{abE\left[X^{i,j}\left(n\right)\right]}{AB-ab}=E\left[X^{i,j}\left(n\right)\right]=K\mu_{Y} \end{array}$$

(A2) $$\begin{array}{ll} D\left[{\bar{X}}^{i_{0},j_{0}}\left(n\right)\right]= & D\left\{\frac{1}{AB-ab}\left[\overset{i_{0}+\frac{A-1}{2}}{\underset{i=i_{0}-\frac{A-1}{2}}{\sum }}\overset{j_{0}+\frac{B-1}{2}}{\underset{j=j_{0}-\frac{B-1}{2}}{\sum }}X^{i,j}\left(n\right)-\overset{i_{0}+\frac{a-1}{2}}{\underset{i=i_{0}-\frac{a-1}{2}}{\sum }}\overset{j_{0}+\frac{b-1}{2}}{\underset{j=j_{0}-\frac{b-1}{2}}{\sum }}X^{i,j}\left(n\right)\right]\right\}\\ & \;={\left(\frac{1}{AB-ab}\right)}^{2}\left\{\overset{i_{0}+\frac{A-1}{2}}{\underset{i=i_{0}-\frac{A-1}{2}}{\sum }}\overset{j_{0}+\frac{B-1}{2}}{\underset{j=j_{0}-\frac{B-1}{2}}{\sum }}D\left[X^{i,j}\left(n\right)\right]-\overset{i_{0}+\frac{a-1}{2}}{\underset{i=i_{0}-\frac{a-1}{2}}{\sum }}\overset{j_{0}+\frac{b-1}{2}}{\underset{j=j_{0}-\frac{b-1}{2}}{\sum }}D\left[X^{i,j}\left(n\right)\right]\right\}\\ & \;=\frac{K\sigma_{Y}^{2}}{AB-ab} \end{array}$$

As for the variance of samples $V^{i_{0},j_{0}}\left(n\right)$ (see (4b)), we notice that

(A3) $$\frac{X^{i,j}\left(n\right)-{\bar{X}}^{i_{0},j_{0}}\left(n\right)}{K\sigma_{Y}^{2}}\sim N\left(0,1\right)$$

thus $\left(AB-ab-1\right)V^{i_{0},j_{0}}\left(n\right)/\left(K\sigma_{Y}^{2}\right)$ is a Chi-square variate with (AB−ab−1) DOFs because (AB−ab) samples $X^{i,j}\left(n\right)$ are mutual independent, which form a single statistics ${\bar{X}}^{i_{0},j_{0}}\left(n\right)$. Moreover, ${\bar{X}}^{i_{0},j_{0}}\left(n\right)$ and $V^{i_{0},j_{0}}\left(n\right)$ are also independent with each other. According to above, conclusions as in (6a) and (6b) are obtained.

Based on (7a) and (7b), it is easy to obtain:

(A4) $${\bar{X}}_{f}^{i_{0},j_{0}}\left(n-1\right)=\omega \overset{n-2}{\underset{l=0}{\sum }}{\left(1-\omega \right)}^{l}{\bar{X}}^{i_{0},j_{0}}\left(n-1-l\right)$$

Let n→∞, such two conclusions are drawn as

(A5) $$E\left[{\bar{X}}_{f}^{i_{0},j_{0}}\left(n-1\right)\right]=\omega \overset{n-2}{\underset{l=0}{\sum }}{\left(1-\omega \right)}^{l}E\left[{\bar{X}}^{i_{0},j_{0}}\left(n-1-l\right)\right]=\frac{\omega K\mu_{Y}}{1-\left(1-\omega \right)}=K\mu_{Y}$$

(A6) $$\begin{array}{cc} D\left[{\bar{X}}_{f}^{i_{0},j_{0}}\left(n-1\right)\right] & =D\left[\omega \overset{n-2}{\underset{l=0}{\sum }}{\left(1-\omega \right)}^{l}{\bar{X}}^{i_{0},j_{0}}\left(n-1-l\right)\right]=\omega^{2}\overset{n-2}{\underset{l=0}{\sum }}{\left(1-\omega \right)}^{2l}D\left[{\bar{X}}^{i_{0},j_{0}}\left(n-1-l\right)\right]\\ & =\frac{\omega^{2}K\sigma_{Y}^{2}}{\left(AB-ab\right)\left[1-{\left(1-\omega \right)}^{2}\right]}=\frac{K\sigma_{Y}^{2}}{\left(AB-ab\right)}\frac{\omega }{2-\omega }=\frac{K\sigma_{Y}^{2}}{\left(AB-ab\right)\varpi } \end{array}$$

Since the PDF of ${\bar{X}}^{i_{0},j_{0}}\left(n\right)$ is Gaussian, ${\bar{X}}_{f}^{i_{0},j_{0}}\left(n\right)\;\sim N\left(K\mu_{Y},K\sigma_{Y}^{2}/\left[\varpi \left(AB-ab\right)\right]\right)$ is easily acquired. ϖ is known as the effective memory length of a filter. Similarly, $V_{f}^{i_{0},j_{0}}\left(n-1\right)$ can be expressed as

(A7) $$V_{f}^{i_{0},j_{0}}\left(n-1\right)=\omega \overset{n-2}{\underset{l=0}{\sum }}{\left(1-\omega \right)}^{l}V^{i_{0},j_{0}}\left(n-1-l\right)$$

It hard to determine the statistical distribution of $V_{f}^{i_{0},j_{0}}\left(n-1\right)$ only by (B-7). Therefore

(A8) $$V_{f}^{i_{0},j_{0}}\left(n-1\right)\approx \frac{1}{\varpi }\overset{\left|\varpi -1\right|}{\underset{l=0}{\sum }}V^{i_{0},j_{0}}\left(n-1-l\right)$$

Based on (6b), $V_{f}^{i_{0},j_{0}}\left(n-1\right)$ can be predicted to obeying a Chi-square distribution with ϖ(AB−ab−1) DOFs as in (8b), which was verified by a test in [29] (p. 250), using 800-population samples. Please note that the Chi-square distribution hypothesis is admitted when 2≤K≤16 and ϖ≥7.

## References

[1] Tarsier^®^. Automatic Runway FOD Detection System. https://www.tarsierfod.com/.

[2] Futatsumori S., Morioka K., Kohmura A. (2016). Design and Field Feasibility Evaluation of Distributed-Type 96 GHz FMCW Millimeter-Wave Radar Based on Radio-Over-Fiber and Optical Frequency Multiplier. J. Lightwave Technol..

[3] AC 150/5220-24—Foreign Object Debris Detection Equipment. https://www.faa.gov/documentLibrary/media/Advisory_Circular/AC_150_5220-24.pdf.

[4] Mazouni K., Zeitler A., Lanteri J. 76.5 GHz millimeter-wave radar for foreign objects debris detection on airport runways. Proceedings of the 8th European Radar Conference.

[5] Mazouni K., Pichot C., Lantéri J. (2012). 77 GHz offset reflectarray for FOD detection on airport runways. Int. J. Microw. Wirel. Technol..

[6] Mazouni K., Kohmura A., Futatsumori S., Yonemoto N., Dauvignac J.-Y., Pichot C., Migliaccio C. 77 GHz FM-CW radar for FODs detection. Proceedings of the 7th European Radar Conference.

[7] Feil P., Menzel W., Nguyen T.P. Foreign objects debris detection (FOD) on airport runways using a broadband 78 GHz sensor. Proceedings of the European Radar Conference.

[8] Mollo G., Napoli R.D., Naviglio G. (2017). Multifrequency Experimental Analysis (10 to 77 GHz) on the Asphalt Reflectivity and RCS of FOD Targets. IEEE Geosci. Remote Sens. Lett..

[9] Galati G., Ferri M., Marti F. Advanced radar techniques for the air transport system: The surface movement miniradar concept. Proceedings of the IEEE National Telesystems Conference.

[10] Galati G., Leonardi M., Cavallin A., Pavan G. (2010). Airport Surveillance Processing Chain for High Resolution Radar. IEEE Trans. Aero Elect. Syst..

[11] Brown A.K. A review of radar as a sensor for advanced surface movement guidance and control systems (A-SMGCS). Proceedings of the IET Aviation Surveillance Systems.

[12] Zhu X., Tang X., Han S. Aircraft Intersection Collision Conflict Detection and Resolution under the Control of A-SMGCS. Proceedings of the 2012 International Conference on Modelling, Identification and Control.

[13] THALES: Discover THALES Research & Technology. https://www.thalesgroup.com/en/global/innovation/research-and-technology.

[14] Conte E., Longo M., Lops M. (1988). Performance analysis of CA-CFAR in the presence of compound Gaussian clutter. Electron. Lett..

[15] Wong C., Chang C., Liu W., Fu J.S. CA-CFAR in Weibull Background. Proceedings of the International Conference on Microwave & Millimeter Wave Technology.

[16] Sciotti M., Lombardo P. Performance evaluation of radar detection schemes based on CA-CFAR against K-distributed clutter. Proceedings of the International Conference on Radar.

[17] Han Y., Kim T. (1996). Performance of excision GO-CFAR detectors in nonhomogeneous environments. IEEE Proc. Radar Sonar Navig..

[18] Jung K.T., Kim H.M. Performance analysis of generalized modified order statistics CFAR detectors. Proceedings of the IEEE-SP International Symposium.

[19] Levanon N. (1988). Detection loss due to interfering targets in ordered statistics CFAR. IEEE Trans. Aero. Elect. Syst..

[20] Di Vito A., Galati G., Mura R. (1994). Analysis and comparison of two order statistics CFAR systems. IEEE Proc. Radar Sonar Navig..

[21] Golikov V., Lebedeva O., Castillejos A. Robust CFAR detection in clutter with unknown covariance matrix. Proceedings of the Computational Advances in Multi-Sensor Adaptive Processing.

[22] Jung C.H., Song W.Y., Rho S.H., Kim J., Park J.T., Kwag Y.K. Double-step fast CFAR scheme for multiple target detection in high resolution SAR images. Proceedings of the IEEE Radar Conference.

[23] Li Y., Wu L., Zhang N. (2018). A CFAR Detector Based on a Robust Combined Method with Spatial Information and Sparsity Regularization in Non-Homogeneous Weibull Clutter. IEEE Access.

[24] Gufran M.H., Thamir R.S., Jafar S. (2018). Comparative Study of Combined CFAR Algorithms for Non-Homogenous Environment. Procedia Comput. Sci..

[25] Nitzberg R. (1986). Clutter Map CFAR Analysis. IEEE Trans. Aero. Elect. Syst..

[26] Meng X. (2010). Performance analysis of Nitzberg’s clutter map for Weibull distribution. Digit. Signal Process..

[27] Lops M. (1996). Hybrid clutter-map/L-CFAR procedure for clutter rejection in nonhomogeneous environment. IEEE Proc. Radar Sonar Navig..

[28] Naldi M. (1999). False alarm control and self-masking avoidance by a bi-parametric clutter map in a mixed interference environment. IEEE Proc. Radar Sonar Navig..

[29] Lops M., Orsini M. (1989). Scan-by-scan averaging CFAR. IEEE Pro. F-Radar Signal Proc..

[30] Conte E., Lops M. (1997). Clutter-map CFAR detection for range-spread targets in non-Gaussian clutter. Part I: System design. IEEE Trans. Aero. Elect. Syst..

[31] Conte E., Bisceglie D.I., Lops M. (2002). Clutter-map CFAR detection for range-spread targets in non-Gaussian clutter. Part II: Performance assessment. IEEE Trans. Aero. Elect. Syst..

[32] Smith M.E., Varshney P.K. VI-CFAR: A novel CFAR algorithm based on data variability. Proceedings of the IEEE Radar Conference.

[33] Smith M.E., Varshney P.K. (2000). Intelligent CFAR processor based on data variability. IEEE Trans. Aero. Elect. Syst..

[34] Cheikh K., Soltani F. Performance of the fuzzy VI-CFAR detector in non-homogeneous environments. Proceedings of the IEEE International Conference on Signal and Image Processing Applications.

[35] Li H., Zhang Z., Wang Y., Yu X. (2016). Performance Analysis and Comparison of CFAR Methods for FOD Detection in Airport Runway Environment. Lect. Notes Electr. Eng..

[36] Li H. (2016). Research about Target Detection in FOD Radar. Master’s Thesis.

[37] Jin E., Yan D., Zhang Z. (2012). FOD Detection on Airport Runway with Clutter Map CFAR Plane Technique. Lect. Notes Electr. Eng..

[38] Wu J., Wang H., Yu X. (2014). CFAR Detection Method in Multi-target Environments for Foreign Object Debris Surveillance Radar. Lect. Notes Electr. Eng..

[39] Wang B., Zhang W. FOD detection based on millimeter wave radar using higher order statistics. Proceedings of the IEEE International Conference on Signal Processing, Communications and Computing.

[40] Mao Y. (2018). Research and Implementation of CFAR Detection Algorithm for Radar Clutter Map. Master’s Thesis.

